# Genome-wide copy number variation regions in indigenous (*Bos indicus*) cattle breeds of Tamil Nadu, India

**DOI:** 10.5713/ab.23.0525

**Published:** 2024-08-26

**Authors:** S. Vani, D. Balasubramanyam, K. G. Tirumurugaan, A. Gopinathan, S. M. K. Karthickeyan

**Affiliations:** 1 Department of Animal Genetics and Breeding, Tamil Nadu Veterinary and Animal Sciences University, Chennai 600 051, India; 2 Current address: Department of Animal Genetics and Breeding, College of Veterinary Science, Proddatur, Sri Venkateswara Veterinary University, Andhra Pradesh 516360, India; 3 Department of Animal Biotechnology, Tamil Nadu Veterinary and Animal Sciences University, Chennai 600 051, India

**Keywords:** *Bos indicus* Cattle, Copy Number Variations, Tamil Nadu, Whole Genome Sequencing

## Abstract

**Objective:**

Identification of large scale structural polymorphisms (copy number variations [CNVs]) of more than 50 bp between the individuals of a species would help in knowing genetic diversity, phenotypic variability, adaptability to tropical environment and disease resistance.

**Methods:**

Read depth-based method implemented in CNVnator was used for calling copy number variant regions on sequenced data obtained from whole-genome sequencing from 15 pooled samples belonging to five draught cattle breeds of Tamil Nadu.

**Results:**

A total of 11,605 CNV regions (CNVRs) were observed covering a genome size of 18.63 percent. Among these, 11,459 were restricted to autosomes, consisting of 11,013 deletions, 353 duplications and 93 complex events. These CNVRs were annotated to 4,989 candidate genes. A total of 8,291 numbers of CNVRs were shared among the five cattle breeds as also supported by principal component analysis and STRUCTURE analyses and 1,172 CNVRs were breed-specific. Four out of five selected breed-specific CNVRs were validated using real-time polymerase chain reaction. Genes with CNVRs are related to milk production (*BTN1A1*, *ABCA1*, and *LAP3*), disease resistance (*TLR4* and *DNAH8*), adaptability (*SOD1*, *CAST*, and *SMARCAL1*), growth (*EGFR*, *NKAIN3*), reproduction (*BRWD1* and *PDE6D*), meat and carcass traits (*MAP3K5* and *NCAM1*) and exterior (*ATRN* and *MITF*) traits. Gene enrichment analysis based on the gene list retrieved from the CNVRs disclosed over-represented terms (p<0.01) associated with milk fat production. NETWORK analysis had identified 13 putative candidate genes involved in milk fat percentage, milk fat yield, lactation persistency, milk yield, heat tolerance, calving ease, growth and conformation traits.

**Conclusion:**

The genome-wide CNVRs identified in the present study produced genome-wide partial CNV map in indigenous cattle breeds of Tamil Nadu.

## INTRODUCTION

India has a massive livestock inventory, with 193.46 million cattle; of which, 51.36 million are exotic/crossbred and 142.11 million are indigenous and non-descript [1]. The latter is remarkably diverse and differentiated into 50 well-defined and registered indigenous cattle breeds that make up the large reservoir of cattle genetic resources, playing a crucial role in augmenting agrarian economy in the country. Of these, Sahiwal, Red Sindhi, Gir, Tharparkar, and Deoni are considered as milch breeds; whereas Ongole, Hariana, Kankrej, Dangi, Rathi, Nimari *etc*. are said to be the major dual purpose breeds; and the rest are considered as draught breeds.

Tamil Nadu, the southernmost state of the country has five draught breeds of cattle (Alambadi, Bargur, Kangayam, Pulikulam, and Umblachery) with distinct phenotypic characteristics. These breeds are reared for their adaptability, draught capacity and disease resistance and also act as prime source of milk to serve the people in the state. Although the cattle population in Tamil Nadu has increased from 7.93 to 8.49 million [1,2], there is a decline in number of indigenous or non-descript population in the state due to indiscriminate cross breeding and swift mechanisation. If this scenario continues over a long term, the population may come under risk category and hence, there is an immense need for preserving genetic variability.

Identification of genetic heterogeneity across cattle breeds is critical for long-term genetic improvement, effective management and conservation of Animal Genetic Resources [3]. Though microsatellites were the markers of choice for breed characterization, parentage testing, analysis of admixture and genetic diversity in different livestock populations for more than two decades [4], but the results obtained are not always comparable due to poor allele size calling [5]. Single nucleotide polymorphism (SNP) markers superseded microsatellites and proved to be highly effective in assessing genetic diversity and population structure of indigenous cattle breeds [6]. Compared with the SNPs, copy number variations (CNVs) can affect a larger portion of the genome and causes change in gene structure, dosage and alters gene regulation.

Currently, there is a dearth of information is available on indigenous cattle breeds at the whole genome level, including sequence variation. Use of whole genome sequencing data for variant discovery has the advantage of reduced SNP ascertainment bias compared to the use of commercially available SNP assays [7]. As on today, the sequence information on whole genome are available only for Nellore cattle of Brazil and Gir cattle of India. Therefore, sequencing the genomes of indigenous cattle could be beneficial in animal production as it identifies variants like SNPs and CNVs at genome level, in understanding the genetic architecture of traits of economic importance, animal health and welfare, genetic basis of diseases, as well as genomic selection-based breeding programs [8].

Copy number variations are large scale structural polymorphisms which include deletions, duplications and insertions of genomic sequences of more than 50 bp between individuals of a species. Unlike SNPs and microsatellites, the population genetics of CNVs is largely unknown [9]. These variations occur less frequently in the genome, but mostly seen in the gene rich areas and are associated with the genetic diversity, regulation of gene expression, phenotypic variability, adaptability to tropical environment and disease resistance [10]. Hence, the present study was carried out to identify the genome-wide CNV regions (CNVRs) in indigenous cattle breeds of Tamil Nadu and also to validate the breed-specific CNVRs.

## MATERIALS AND METHODS

### Ethical approval

The present study does not warrant permission from Institutional Animal Ethics Committee as the study was carried out in “AS IS” condition in their natural habitat without any experimentation performed on them.

### Sample preparation and whole genome sequencing

A total of 302 representative samples from five draught cattle breeds of Tamil Nadu were utilized in the present study. An amount of 3 mL of blood sample was collected aseptically from the jugular veins from each animal viz. Alambadi (16), Bargur (106), Kangayam (63), Pulikulam (56), and Umblachery (61) cattle. Care was taken to avoid close relationship among them and possible phenotypic variants within the breed were covered while sampling. Genomic DNA was isolated using standard phenol-chloroform extraction procedure recommended by Sambrook [11] with slight modifications by using DNAzol reagent, instead of sodium dodecyl sulphate and proteinase K. All DNA samples were analyzed by spectrophotometry and agarose gel electrophoresis.

Of the total samples, 79 DNA samples were selected and pooled into bulls, moderately yielding dams and low yielding dams under each breed as furnished in Supplementary Table S3. The concentration of DNA from selected individuals has been properly balanced to 200 ng/μL with TE buffer in order to represent each genome equally [12]. Equal volume of DNA (10 μL) from each selected individual of respective group were combined to obtain group-specific pools, that would give enough DNA for subsequent process of whole genome sequencing. Thus a sum of 79 samples from five breeds pooled into 15 groups (ACG1, ACG2, and ACG3 for Alambadi; BCG1, BCG2, and BCG3 for Bargur; KCG1, KCG2, and KCG3 for Kangayam; PCG1, PCG2, and PCG3 for Pulikulam; and UCG1, UCG2, and UCG3 for Umblachery cattle) were prepared and sequenced using paired-end libraries on Illumina Hiseq 2500 and Novoseq 6000 platforms.

### Quality filtering of raw sequence reads and alignment

The sequence data was checked for base call quality distribution, phred score quality (Q score), GC percentage and sequencing adapter contamination using FastQC [13] and MultiQC [14] software. The fastp v0.20.1 was used to trim adapter sequences and low quality bases with a phred score of less than 20. All the reads were then adjusted by trimming to 151 bp to omit the error-prone ends. The processed reads were mapped against *Bos indicus* reference genome (GCF_ 000247795.1_Bos_indicus_1.0) using BWA-MEM algorithm v0.7.17-r1188 with the default parameters. The obtained SAM file was converted into BAM file using SAMtools. The resultant BAM files underwent sorting and filtering for duplicates using PICARD tool v 2.0.1.

### Identification of copy number variation regions

CNVnator v 0.4.1 tool, a read depth approach was employed for the merged BAM files of each pooled cattle sample associated with the reference genome (GCF_000247795.1_Bos_indicus_1.0) to identify CNVs using default parameters [15]. Venn diagram was plotted to represent the CNVRs shared among cattle breeds using Venn Diagram package in R platform. Ideogram was constructed to show the total number of CNVRs and breed-specific CNVRs of the present study using karyoploteR package in ‘R’ platform.

### Gene detection and quantitative trait loci mapping

Genes partially harboured or overlapped within the detected CNVRs were identified across all the five cattle breeds using SnpEff v 5.0e tool. Each CNVR was annotated using.gff (general feature format) annotation file of the *Bos indicus* reference genome. Since QTLdb does not exist for *Bos indicus* reference genome, information on trait-associated genes was obtained from previous reports and also from animal QTLdb (https://www.animalgenome.org/cgi-bin/QTLdb/BT/index) of *Bos taurus* genome to analyze important associations among the identified CNVRs and economically important traits in draught cattle of Tamil Nadu.

### Population structure analysis

Principle component analysis was performed using plink v1.90b6.21 and plot was generated using the ggplot2 package in ‘R’ platform. Admixture analysis of the identified CNVRs was carried out using STRUCTURE v 2.3.4 to reveal the degree of admixture between indigenous cattle breeds of Tamil Nadu. The Admixture model employed in STRUCTURE without the LocPrior option was used, with a 5,000 burning period and 10,000 iterations, performing five repeats for each K value from 1 to 5 and assuming five different populations.

### Functional annotation and enrichment analysis

Gene ontology (GO) categories (biological processes, molecular functions, cellular components) and Kyoto encyclopedia of genes and genomes (KEGG) pathway analyses were determined using Database for Annotation Visualization and Integrated Discovery (DAVID knowledgebase v.2021) functional annotation tool (https://david.ncifcrf.gov/list.jsp) and uniprotKB for the genes containing the CNVRs with identified phenotypes. Only the enriched GO terms with raw p-values <0.05 after a Benjamini-Hochberg multiple testing correction were used for further interpretation in this study.

### Network analysis

Inter-relationship between the candidate genes identified for various phenotypes was tested using NETWORK analyst v 3.0 online tool for different GO terms using string first order interactome database with a confidence score cut-off of 900.

### Quantitative polymerase chain reaction validation of breed-specific copy number variation regions

Quantitative polymerase chain reaction (qPCR) was used to validate CNVRs detected by CNVnator on a total of 84 samples (79 samples used for pooling and five more samples were taken additionally) viz. Alambadi (15); Bargur (22); Kangayam (17); Pulikulam (16); Umblachery (14) to find the relative fold change in copy numbers. A total of five breed-specific genic CNVRs overlapped with *PRKG1*, *GLDC*, *RERE*, *KIF11*, and *FBXO40* genes that are known to influence tick resistance; milk production; fertility traits were chosen. Along with these five genes, basic transcription factor 3 (*BTF3*) gene was considered as the internal control gene with the assumption that there were two copies of DNA segment in this gene [16]. For each genic CNVR, as well as *BTF3* gene, best primers were determined after designing multiple pairs of primers due to uncertainty of the CNVR boundaries using Primer3 web tool. The details of primers used in the present study along with their genomic locations are furnished in Supplementary Table S4. Standard curve was plotted to ensure the amplification efficiencies of all pairs of primers for which, a serially diluted genomic DNA from a common cattle was used as template. For relative quantification, qPCR experiment was performed using SYBR green chemistry in duplicate reactions using Bio-Rad thermocycler, each with a reaction volume of 10 μL. Conditions for real-time PCR included initial denaturation for 3 min at 95°C followed by 40 cycles of two-step RT-PCR (denaturation for 25 s at 95°C and annealing, elongation for 45 s at 63°C). To reduce batch and platform effects, plates were designed to amplify the reference gene and a pooled sample without CNVR as confirmed by whole-genome sequencing (WGS) in each experiment. For each CNVR to be validated, the value of 2×2^−ΔΔCt^ was calculated for each individual [17]. First, we obtained the average Ct value of two replications of each sample and normalized against the control gene. Then we calculated the ΔCt value between the test sample and reference sample detected with normal status (i.e. two copy numbers) by CNVnator. The obtained value was used to decide if a CNVR was normal (if the value was about two) or duplication (if the value was about three or above) or deletion (if the value was near zero or one).

## RESULTS

### Whole genome sequencing data statistics

With Illumina paired-end sequencing technology, NGS data for 15 pooled samples were obtained. After mapping them on the GCF_000247795.1_*Bos_indicus*_1.0 bovine genome assembly and excluded potential PCR duplicates, the average depth of coverage across all samples was found to be 19.11x.

### Landscape of identified genome-wide copy number variation regions

**Figure 1:**
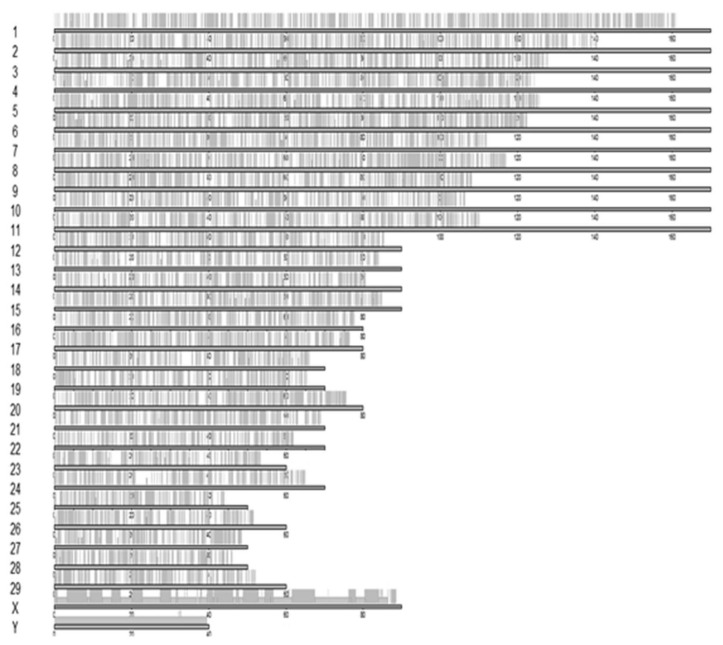
Ideogram showing location of copy number variation region (CNVR) types in cattle breeds of Tamil Nadu.

A total of 11,605 CNVRs were observed among all animals (n = 15; pooled samples belonging to 79 animals of five draught cattle breeds of Tamil Nadu) using CNVnator tool. Chromosome-wise summary statistics of CNVRs across cattle breeds of Tamil Nadu is presented in Supplementary Table S1. Chromosome one contained the highest number of CNVRs (737) covering a length of 20,927,000 bp with a mean of 28.39 kb per CNVR. While, Y chromosome contained the lowest number (12) covering a length of 39,177,000 bp with a mean of 3,264.75 kb per CNVR. The correlation between the number of detected CNVRs and the chromosome size was 0.85. Percentage length of CNVRs with respect to their chromosome size ranges from 9.65 (chromosome 22) to 99.38 (chromosome Y) with an average of 16.85 across all chromosomes. Total number of CNVRs along with their percentage length in each chromosome are depicted in Supplementary Figure S1. Figure 1 showed the ideogram of location of CNVR types in cattle breeds of Tamil Nadu. Since, CNVRs in allosomes accounted for 97.02 (X) and 99.38 (Y) percent of their respective chromosome lengths, these were removed from the further downstream analysis.

**Figure 2:**
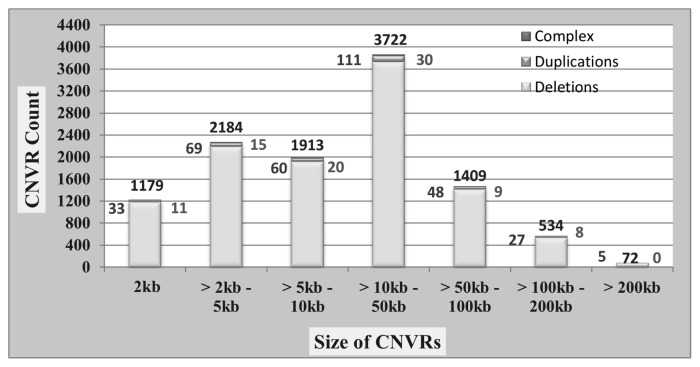
Size distribution of copy number variation regions (CNVRs) in cattle breeds of Tamil Nadu.

**Figure 3:**
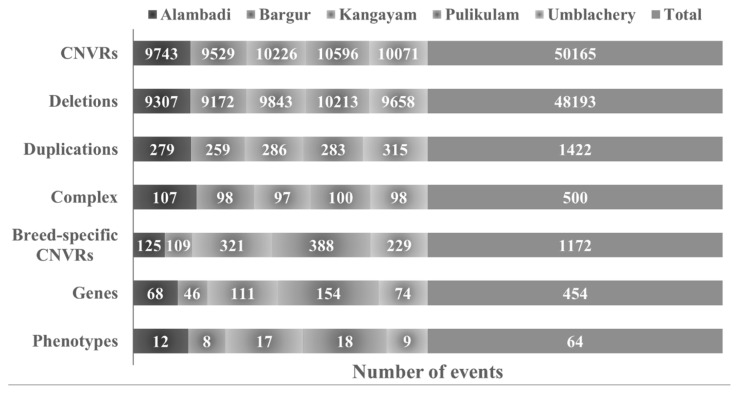
Breed-wise profile of copy number variation regions (CNVRs). Stacked bar chart showing the total number of CNVRs, deletions, duplications, complex events, breed-specific CNVRs, breed-specific genic CNVRs annotated and phenotypes studied.

After exclusion of CNVRs present on sex chromosomes, a total of 11,459 CNVRs were identified among all animals. Among these, 11,013 CNVRs were deletions (losses), 353 were duplications (gains) and 93 were complex events (both). Highest numbers of CNVRs were reported in Pulikulam cattle breed (10,213) while the lowest (9,172) in Bargur. The ratio of CNVRs loss to gains was 31.20:1. The total length of the detected CNVRs across breeds ranged from 312.225 Mb (Kangayam) to 475.118 Mb (Bargur) with a mean total length of 325.558 Mb, and coverage of 12.058 percent with respect to their genome size. The size of CNVRs as shown in Figure 2 varied from 2 to 572 kb with an average of 28.42 kb. Table 1 summarized the characteristics of CNVRs in indigenous cattle breeds of Tamil Nadu. Breed-wise profile of the total CNVRs, deletions, duplications, complex events, breed-specific CNVRs, number of genes affected and phenotypes studied for each breed are represented in Figure 3.

### Breed-shared and breed-specific copy number variation regions

**Figure 4:**
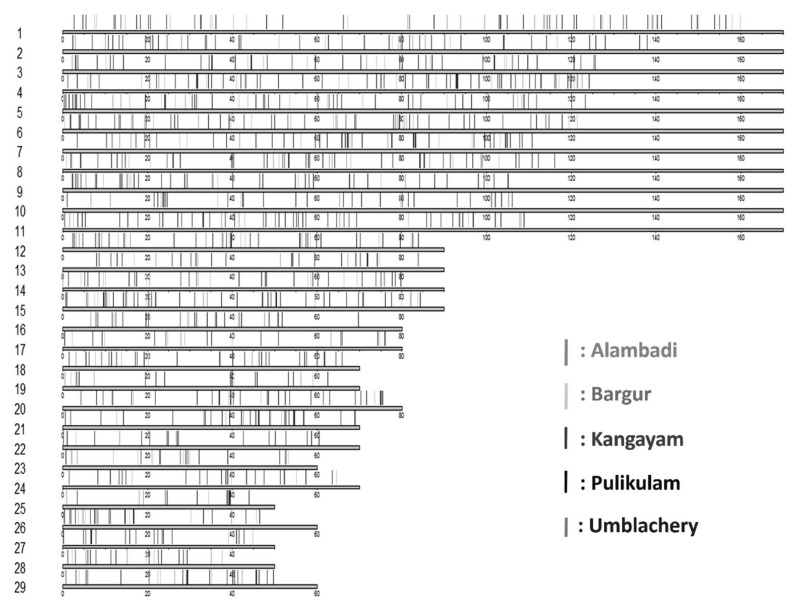
Distribution of breed-specific copy number variation regions (CNVRs) across chromosomes.

**Figure 5:**
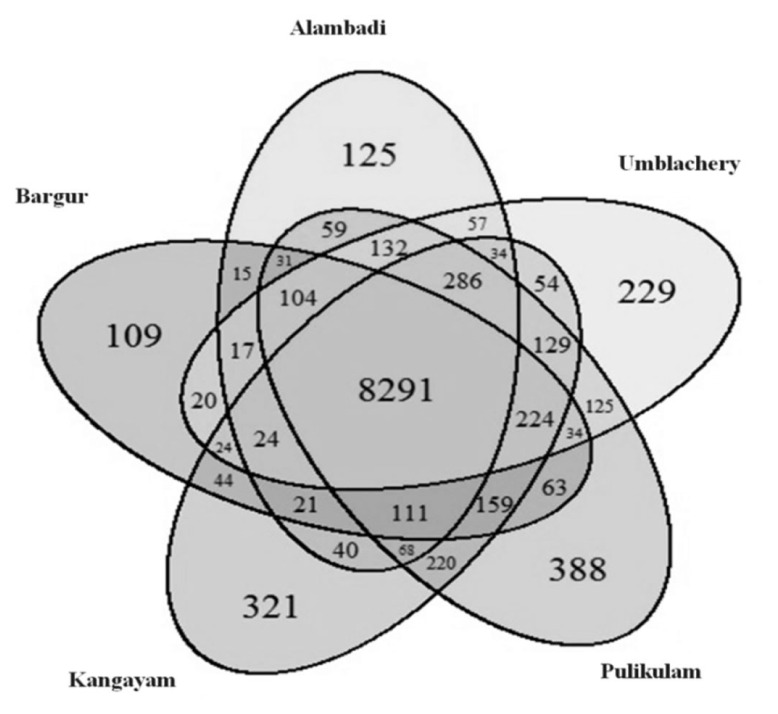
Venn diagram showing overlap as well as breed-specific copy number variation regions (CNVRs) across cattle breeds of Tamil Nadu.

A number of CNVRs were shared between breeds. Out of 11,459 CNVRs identified across the genome, 8,291 numbers of CNVRs were observed to be shared among the five cattle breeds indicating high degree of admixture between them. Of these, 7,946 were deletions, 194 were duplications and 151 were complex events. A total of 1,172 number of CNVRs were only identified specifically in one or the other breeds as shown in Figure 4. Pulikulam had the highest number (388) of breed-specific CNVRs followed by Kangayam (321), Umblachery (229), Alambadi (125), and Bargur (109). List of CNVRs shared between any five, four, three, or two cattle breeds of Tamil Nadu and breed-specific CNVRs are presented in Table 2. Diagramatic representation of these CNVRs plotted as a venn diagram is shown in Figure 5.

### Population structure analysis

The first two principal components (PC_1_ and PC_2_) of the principal component analysis (PCA) shown in Supplementary Figure S2 based on CNVR presence or absence data explained 16.37 and 10.03 percent of the total variability of data respectively. Similar results were observed for CNV deletion and duplication data (Supplementary Figure S3) where 15.96 and 10.22 percent of the total genetic variation was explained by PC_1_ and PC_2_ respectively. PC_1_ and PC_2_ explained about nearly 26 percent of the total variation in both data sets. All the indigenous breeds of Tamil Nadu did not separate clearly from each other. Umblachery, Alambadi, and low yielders of Pulikulam breed formed a separate cluster, distinct from the Bargur, Kangayam, and Pulikulam (bulls and medium yielders) showing the highest genetic distance between them. The admixture analysis did not reveal any detectable population substructure. Structure bar plot showing admixture of indigenous cattle breeds of Tamil Nadu at different ‘k’ values is illustrated in Supplementary Figure S4.

### Gene detection and quantitative trait loci mapping

**Figure 6:**
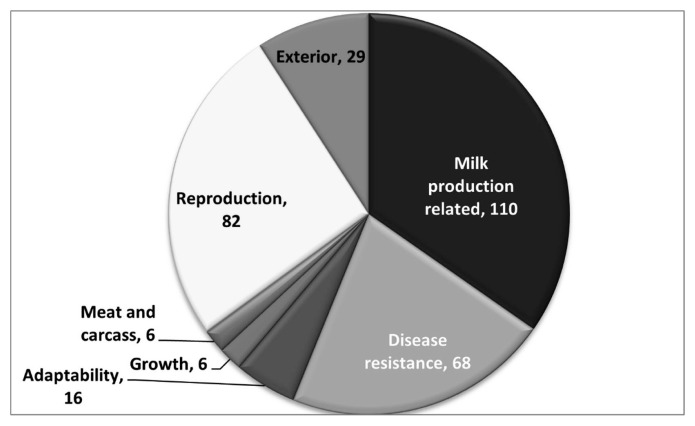
Number of quantitative trait locus identified in cattle breeds of Tamil Nadu.

A total of 4,989 candidate genes had been found within the 11,459 CNVRs across all the five cattle breeds using SnpEff v 5.0e tool. A total of 317 out of 4,989 genes in CNVRs were successfully mapped to the quantitative trait loci (Supplementary Table S2). These genes were categorized into seven categories viz. milk production related (110), health (68), adaptability (16), growth (6), meat and carcass (6), reproduction (82), and exterior traits (29) as detailed in Figure 6.

### Functional annotation of candidate genes bearing copy number variation regions

#### Gene ontology terms

Gene enrichment analysis done using DAVID functional annotation tool identified 198 genes (73 genes with cellular components, 55 genes with biological processes and 70 genes with molecular functions) were over-represented with p<0.05 and these were related to cellular processes and maintenance, intra-cellular signal transduction, lipid and fatty acid metabolism, phosphotidyl inositol binding, calcium activity and neuro-developmental processes as detailed in Supplementary Figure S5. List of genes involved in various KEGG pathways are presented in Table 3.

#### Network analysis

**Figure 7:**
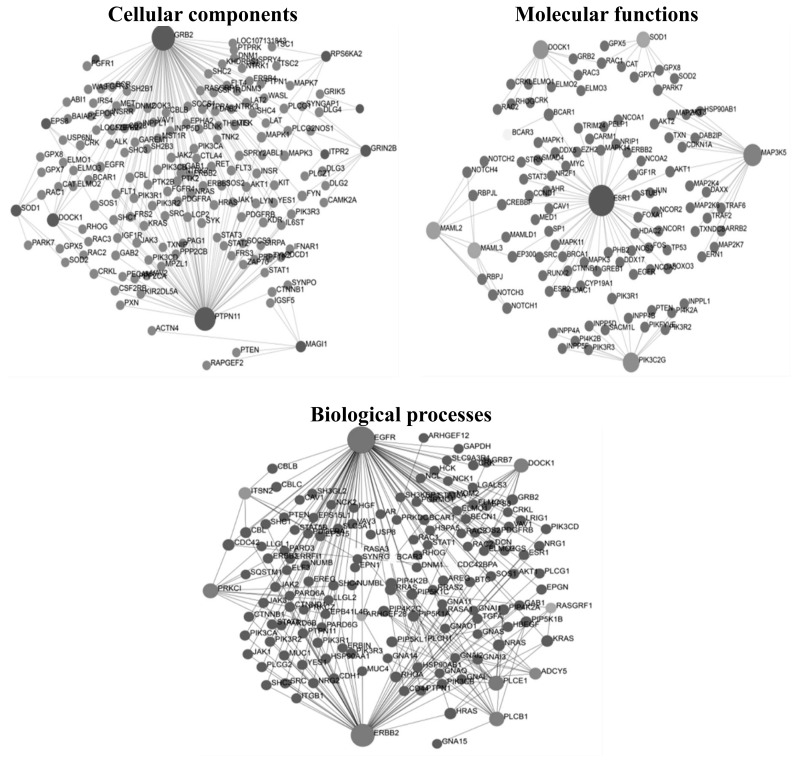
Gene network analysis for the prioritized candidate genes containing copy number variations (A) cellular components, (B) molecular functions and (C) biological processes. The big circles represent prioritized genes, while the small circles denote the related genes.

Network analysis constructed for functional GO network built interrelated terms based on the prioritized genes shared between GO terms using Network Analyst v.3.0. Networks with nodes and edges were generated, and the networks with GO terms such as molecular functions, biological processes, and cellular components were also produced and portrayed in Figure 7. This had identified 13 putative candidate genes involved in milk fat percentage (*ERBB2*, *PLCE1*, and *PIK3C2G*), milk fat yield (*PTPN1*), lactation persistency (*MAP3K5*), milk yield (*MAML2*), heat tolerance (*SOD1*), calving ease (*DOCK1*, *MAML3*, *PLCB1*, and *ESR1*), growth (*EGFR*) and conformation traits (*GRB2*).

### Validation of breed-specific copy number variation regions

CNVR validation and its accuracy were measured by real time PCR. Four of the five (three deletion events in *PRKG1*, *RERE*, and *FBXO40*, and two duplication events in *GLDC* and *KIF11* genes) selected CNVRs (80 percent) successfully exhibited the expected copy number differences in the pooled DNA samples of five cattle breeds of Tamil Nadu. One CNVR located on Chr.16 overlapped with the *RERE* gene, a region of the genome that corresponds to the fat production in cattle, could not be validated. It showed both deletion and duplication instead of only deletion reported in WGS. Validation report of CNVRs obtained using whole genome sequencing in pooled samples in comparison to *BTF3* reference gene of indigenous cattle breeds of Tamil Nadu are detailed in Table 4. CNVRs were also validated for individual samples present in each pooled group and most of them were in accordance with pooled samples with few exceptions. Out of 84 samples screened, the overall matching rate of the CNVRs ranged from 60.71 (*PRKG1*) to 92.86 (*GLDC*) percent with an overall average of 77.86 percent, which was considered to be the CNV accuracy.

## DISCUSSION

### Sample preparation and whole genome sequencing

Pooling of samples strengthened the richness of CNVR calling in the present study as also reported in Gir cattle breed of Brazil [18]. With an average depth rate of 19.11× in the present study, the CNVRs were called with high accuracy as an average depth between 4 and 8× allowing sufficient power for CNV detection using the read depth-based method [19]. This was higher than that reported for Mangolian (13.90×) and Minnan (12.80×) cattle breeds [20].

### Copy number variation region analysis

The correlation coefficient between number and size of chromosomes (0.85) was found to be relatively low in comparison to that reported in the Nellore cattle [21]. After exclusion of CNVRs present on sex chromosomes, there were 11,459 CNVRs on autosomes. These results were in accordance with the reports of Liu et al [22], where, 11,486 CNVRs across autosomes in 75 individuals were obtained using arrayCGH approach. Stothard et al [23] and Zhang et al [24] reported 790 CNVRs in two *Bos taurus* bulls, and 486 CNVRs in 24 taurine, 161 CNVRs in two Yak and 163 CNVRs in three buffaloes respectively, which were very low compared to the present study. On the other hand, in separate studies conducted on Nellore cattle using *Bos taurus* as reference genome, da Silva et al [21] and Zhou et al [25] reported extensively high number of CNVs than the present study with values of 68,007 and 992,350 respectively.

The proportion of the genome covered by these autosomal CNVRs (12.058 percent in the present study) is in agreement with that reported from a meta data taken from bovine HapMap SNP genotyping [26]. Whereas, previous studies revealed lower proportion of CNVRs in the genome as 1.07, less than one and 7.47 percent respectively [23,25,27]. The discrepancy observed in the number, size and proportion of CNVRs in the genome observed in different studies is attributable to differences in number of samples used for analysis, reference genome used for mapping, the bio-informatic tool applied for identifying the CNVs and CNVRs, and also closeness of breeds used for analysis.

The number of deletions, duplications and complex events of the present study are at par with results of Liu et al [27] where, a total of 10,853 losses, 531 gains and 102 complex events were reported. The ratio of deletion to duplications in the present study was comparatively higher than that reported in other NGS studies by several researchers *viz*. Gao et al [15] and Liu et al [22] who reported the values as 1.72 and 20.44 respectively for different taurine cattle breeds. Eventhough there were less duplications, they supplied extra copies of the gene, which would increase the flexibility of gene loss due to selection pressure [22].

### Population genetic structure analysis

The PCA using CNVR data, placed Bargur and Kangayam in a sub-cluster which could be due to geographical proximity between them. Presence of more number of overlapping CNVRs (8,291 numbers) in all the five cattle breeds is the reason for failure of stratification of the breeds. This clearly suggests that a portion of the genome containing CNVRs has been highly conserved in indigenous cattle population of Tamil Nadu. Further, the admixture noticed in the present study affirm that, these cattle breeds of Tamil Nadu have diverged from common ancestral population and are primarily reared for their draught ability; and the selection practised in these breeds, might have generated variants of common importance, as opined by Manomohan et al [28] in draught cattle breeds of South India.

### Breed-shared and breed-specific copy number variation regions

CNVRs shared between breeds in the current investigation suggest that, an important portion of the genome containing CNVRs was conserved within the indigenous cattle populations of Tamil Nadu.

Breed-specific CNVRs contribute for the differences in adaptation, health, and production traits between the breeds. Due to still relatively small sizes of breed-specific data sets in the present as well as previous NGS based studies, an unequivocal declaration of a CNV being specific for only one breed is not possible. The result of the present exploration (1,172) was comparatively less than that reported in Chinese (5,146), Leiqiong (384), Japanese Black (249), and Red Angus (470) cattle breeds [22]. Similarly, 1,164 and 596 breed-specific CNVs were identified in Minnan and Mongolian cattle breeds respectively [20]. While, comparable estimates of 767 and 187 breed-specific CNVs were reported in Hanwoo and Holstein cattle respectively [29].

### Functional annotation and enrichment analysis

Similar GO terms were also reported in dairy cattle and Indian buffaloes respectively [30,31]. Several important signalling pathways related to milk production (calcium, oxytocin), reproduction (GnRH, estogen, relaxin), growth (MAPK signalling) and metabolism were enriched (p<0.05).

MAPK signalling pathway (*HGF*, *CACNA2D1*, *RASGRF1*, *PLA2G4A*, *CACNA1D*, *EGFR*, *RASGRP3*, *CACNB2*, *PPM1B*, *RPS6KA2*, *ERBB2*, *FLNB*, *GRB2*, *MAP3K5*, and *MAP4K4*) is responsible for cell proliferation and plays an important role in hyper plastic growth (Chang, 2007), which has a positive effect on meat tenderness [32]. It is also important in gap junctions, regulation of actin cytoskeleton and residual feed intake.

Calcium signalling pathway (*SLC8A3*, *EDNRB*, *STIM1*, *HGF*, *CHRNA7*, *PDE1A*, *ERBB2*, *PLCE1*, *ITPR2*, *CACNA1D*, *PLCB1*, and *EGFR*) has been reported to be the key pathway for thermal sweating [33,34]. Oxytocin signalling pathway regulates the milk flow [35] and estrogen signalling pathway stimulates bone formation [36,37].

All these prioritization analyses such as GO, KEGG and Network, could identify key candidate genes associated with milk fat production, disease resistance, heat tolerance, and adaptation to tropical climates in the cattle breeds of Tamil Nadu. The present investigation highlighted the importance of *Bos indicus* cattle, which perform better in terms of both production (fat percent) and adaptation to tropical climate due to their unique genetic mechanism that were deciphered. The future looks more promising since the implementation of these genomic information might enhance the genetic progress in cattle breeds of Tamil Nadu.

### Quantitative trait loci mapping

A total of 11,459 CNVRs identified in the present were compared with the reported QTLs from the cattle QTL database as well as previous reports and identified a total of 317 QTLs in seven classes of traits. Indigenous cattle of Tamil Nadu are an essential source of draft energy and are also a considerable source of milk for the people in the state. The most common production trait associated with QTLs was milk fat percentage, milk protein percentage and milk yield. Buaban et al [35] reported similar QTL for milk solids in their study. This indicates the importance of these Tamil Nadu cattle as the primary source for milk production. The majority of health-related QTLs were linked to bovine respiratory disease susceptibility and resistance to mastitis. Some QTLs associated with heat tolerance, regulation of body temperature, oxidative stress response were also identified in these cattle breeds which support their heat tolerance capacity to hot climatic conditions of the state. Most of the QTLs associated with calving ease were also identified.

### Validation of breed-specific copy number variation regions

The high success rate of the real time-PCR validation experiment shows that most of the detected CNVRs in this study are reliable, mainly because of the strict criteria that were set-up to detect the CNVRs. Similar results were reported by Choi et al [38] where nine out of 11 (81.82 percent) CNVRs were successfully validated in Hanwoo, Black Angus, and Holstein cattle using real-time PCR. Liu et al [27] reported higher percentage of CNVR validation (11 out of 12; 91.67 percent) in his findings in 17 diverse cattle breeds than the present studywhile, Stothard et al [23] validated all the 10 selected CNVRs (100 percent) using the real-time PCR. On the contrary, Jiang et al [39] validated only six out of 12 selected variable regions (50 percent) using real-time PCR which is less compared to the current study. While, Shin et al [29] examined 19 CNVRs in 10 Holstein and 22 Hanwoo cattle and reported varied matching rates between the observed and expected CNVRs ranging from 37.19 to 100 percent. These extensive variations within a breed and between breeds are not unexpected as CNVRs detected in this study are based on 15 pooled DNA samples from five cattle breeds. It is well recognized that limited sample size, ethnic diversity of CNVR distribution, and technology/platform employed (CNVnator) could substantially affect the identification of potential CNVRs, including novel and common CNVRs [40].

## CONCLUSION

The genome-wide CNVRs in five draught cattle breeds of Tamil Nadu were presented. Functional enrichment analyses of the genes identified within these CNVRs revealed several potential biological processes that answered the adaptability and growth traits of indigenous cattle. QTLs analyses showed that many of the CNVRs overlapped with QTLs are linked with economically important traits in cattle. As this is a preliminary report, we further suggest whole-genome sequencing from different indigenous cattle breeds with larger sample size to be done to develop an inclusive genome-wide indigenous cattle CNV map.

## Figures and Tables

**Table 1:** Genomic characteristics of CNVRs in indigenous cattle breeds of Tamil Nadu

Breed	No. of samples pooled	CNVRs	Length (Mb)	Loss (No.)	Gain (No.)	Complex (No.)	Coverage (percent)	Mean CNVRs per animal
Alambadi	14	9,307	312.337	8,971	268	68	11.568	664.79
Bargur	19	9,172	475.118	8,865	248	59	17.597	482.74
Kangayam	16	9,843	312.225	9,507	275	61	11.564	615.19
Pulikulam	16	10,213	352.669	9,876	270	67	13.062	638.31
Umblachery	14	9,658	346.388	9,296	267	95	12.829	689.86
Merged/total	79	11,459	325.558	11,013	353	93	12.058	145.05

CNVRs, copy number variation regions.

**Table 2:** Breed-wise overlap of CNVRs

Breeds	No. of CNVRs	Deletions	Duplications	Complex
All five breeds^1)^	8,291	7,946	194	151
Any four breeds				
A, B, K, P	111	102	8	1
B, K, P, U	224	217	6	1
K, P, U, A	286	275	11	-
P, U, A, B	104	98	5	1
U, A, B, K	24	22	2	-
Any three breeds				
A, B, K	21	17	4	-
A, B, P	31	27	4	-
A, B, U	17	16	1	-
A, K, P	68	67	-	1
A, K, U	34	33	1	-
A, P, U	132	125	7	-
B, K, P	159	155	4	-
B, K, U	24	23	1	-
B, P, U	34	34	-	-
K, P, U	129	127	1	1
Any two breeds				
A, B	15	14	1	-
A, K	40	39	-	1
A, P	59	58	1	-
A, U	57	55	2	-
B, K	44	41	3	-
B, P	63	63	-	-
B, U	20	20	-	-
K, P	220	219	1	-
K, U	54	50	4	-
P, U	125	122	3	-
Breed-specific CNVRs				
Alambadi	125	112	13	-
Bargur	109	99	10	-
Kangayam	321	308	13	-
Pulikulam	388	371	16	1
Umblachery	229	214	15	-

CNVRs, copy number variation regions.

1) A, Alambadi; B, Bargur; K, Kangayam; P, Pulikulam; U, Umblachery.

**Table 3:** List of genes involved in KEGG pathways

Sl.No	KEGG Pathway	No.of genes	Genes involved
1	Oxytocin signalling pathway	12	*GUCY1A2, CACNB2, CDKN1A, CACNA2D1, ITPR2, PLA2G4A, CACNA1D, PLCB1, PRKAG3, EGFR, ADCY5 and KCNJ3*
2	MAPK signalling pathway	15	*HGF, CACNA2D1, RASGRF1, PLA2G4A, CACNA1D, EGFR, RASGRP3, CACNB2, PPM1B, RPS6KA2, ERBB2, FLNB, GRB2, MAP3K5 and MAP4K4*
3	Calcium signalling pathway	12	*SLC8A3, EDNRB, STIM1, HGF, CHRNA7, PDE1A, ERBB2, PLCE1, ITPR2, CACNA1D, PLCB1 and EGFR*
4	Ras signalling pathway	12	*RASA3, HGF, RASGRF1, PLCE1, PLA2G4A, PTPN11, GRB2, KSR2, GRIN2B, PAK5, EGFR and RASGRP3*
5	Rap1 signalling pathway	11	*MAGI1, PRKCI, HGF, PLCE1, PRKD1, DRD2, PLCB1, GRIN2B, EGFR, ADCY5 and RASGRP3*
6	GnRH signalling pathway	7	*ITPR2, PLA2G4A, CACNA1D, GRB2, PLCB1, EGFR and ADCY5*
7	ErbB signalling pathway	6	*CDKN1A, NRG3, ERBB2, GRB2, PAK5 and EGFR*
8	cGMP-PKG signalling pathway	8	*SLC8A3, GUCY1A2, EDNRB, KCNMA1, ITPR2, CACNA1D, PLCB1 and ADCY5*
9	JAK-STAT signalling pathway	8	*CDKN1A, IL7, IL2RB, AOX1, PTPN11, GRB2, IL2 and EGFR*
10	Metabolic pathways	37	*ACSS3, PDE1A, GADL1, MGST1, PIK3C2G, HSD17B12, CSAD, FHIT, ADCY5, FADS2, PDE11A, MAN2A1, PRDM16, PDE6D, PLCE1, AOX1, ASL, LTA4H, GALNT8, CSGALNACT1, GUCY1A2, ST8SIA1, ELOVL5, SCD5, PLA2G4A, ELOVL7, DERA, PCCA, DPYD, TBXAS1, SUCLG2, PLCH1, LAP3, ACO2, PLCB1, LPIN1 and PDE9A*
11	Relaxin signalling pathway	6	*EDNRB, COL4A6, GRB2, PLCB1, EGFR and ADCY5*
12	FoxO signalling pathway	6	*CDKN1A, BCL6, TNFSF10, GRB2, PRKAG3 and EGFR*
13	Estrogen signalling pathway	6	*ITPR2, GRB2, PLCB1, EGFR, ADCY5 and KCNJ3*

KEGG, Kyoto encyclopedia of genes and genomes.

**Table 4:** Validation report of CNVRs obtained in whole genome sequencing in pooled samples of indigenous cattle breeds of Tamil Nadu

CNVR	Chr. No.	Breed	CNVR reported in WGS	CNVR obtained in real-time polymerase chain reaction	Remarks
*BTF3*	20	All breeds	Normal	Normal	Validated
*PRKG1*	26	Alambadi	Deletion	Deletion	Validated
*GLDC*	8	Bargur	Duplication	Duplication	Validated
*RERE*	16	Kangayam	Deletion	Deletion and Duplication	Not Validated
*KIF11*	26	Pulikulam	Duplication	Duplication	Validated
*FBXO40*	1	Umblachery	Deletion	Deletion	Validated

CNVRs, copy number variation regions.

## SUPPLEMENTARY MATERIAL

**Supplementary Figure S1.** Chromosome-wise number and percentage length of CNVRs in cattle breeds of Tamil Nadu.

**Supplementary Figure S2.** Principal component analysis of indigenous cattle breeds of Tamil Nadu based on CNVR presence and absence data. **Supplementary Figure S3.** Principal component analysis of indigenous cattle breeds of Tamil Nadu based on deletions and duplications of CNV regions.

**Supplementary Figure S4.** Structure bar plot showing admixture of indigenous cattle breeds of Tamil Nadu (K = 2 to 5).

**Supplementary Figure S5.** Gene ontology terms of candidate genes.

**Supplementary Table S1.** Chromosome-wise summary of CNVRs across cattle breeds of Tamil Nadu

**Supplementary Table S2.** Annotation of CNVRs in candidate genes for various phenotypic traits

**Supplementary Table S3.** Details of criteria followed for pooling of DNA samples infive cattle breeds of Tamil Nadu

**Supplementary Table S4.** Details of primers used for validation of copy number variation regions
