# Supplementary material for: Genome-wide copy number variation regions in indigenous (Bos indicus) cattle breeds of Tamil Nadu, India
Source: Anim Biosci. 2024 Aug 26;38(3):395–407. doi: 10.5713/ab.23.0525 (PMC11917407; doi:10.5713/ab.23.0525)
Supplement: Supplementary file 1 [file ab-23-0525-Supplementary-Fig-S1.pdf]

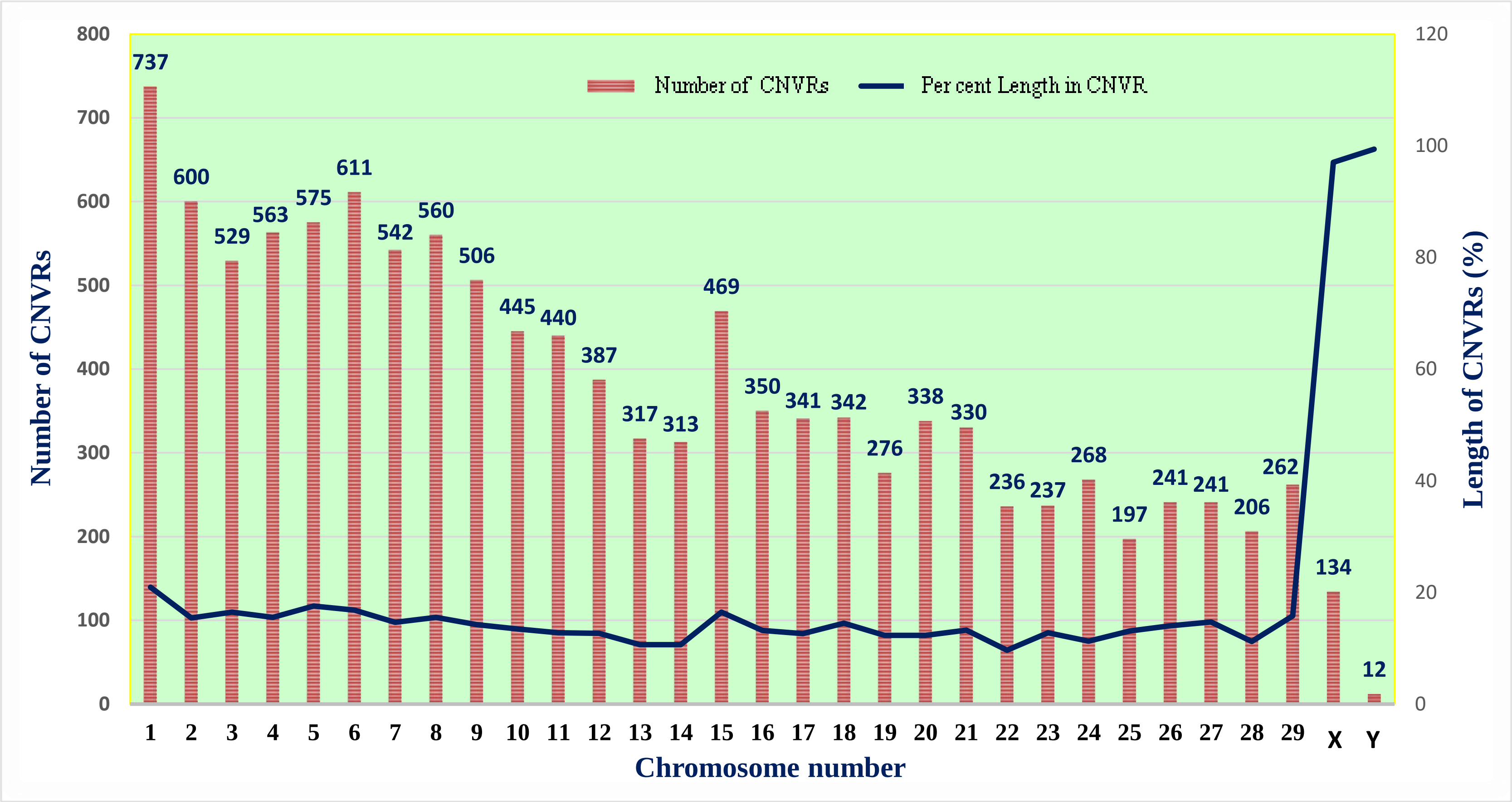

Supplementary Fig. 1. Chromosome-wise number and percentage length of CNVRs in cattle breeds of Tamil Nadu
