# Supplementary material for: Genome-wide copy number variation regions in indigenous (Bos indicus) cattle breeds of Tamil Nadu, India
Source: Anim Biosci. 2024 Aug 26;38(3):395–407. doi: 10.5713/ab.23.0525 (PMC11917407; doi:10.5713/ab.23.0525)
Supplement: Supplementary file 2 [file ab-23-0525-Supplementary-Fig-S2,3.pdf]

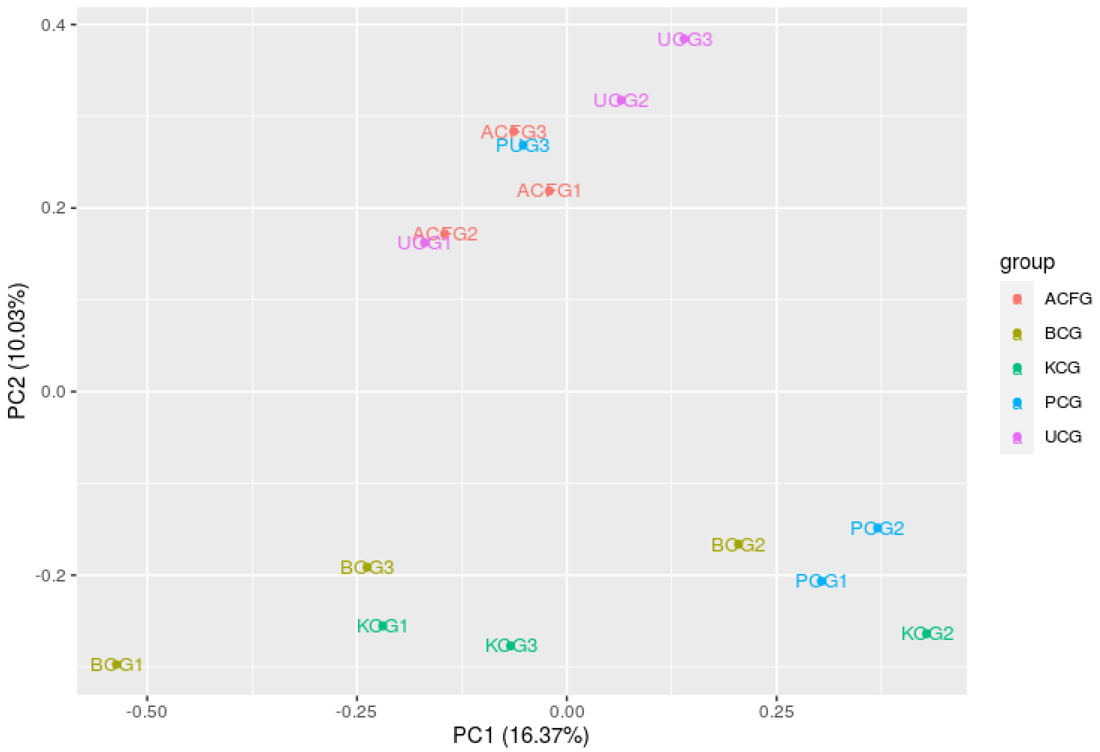

Supplemntary Fig. 2. Principal component analysis of indigenous cattle breeds of Tamil Nadu based on CNVR presence and absence data

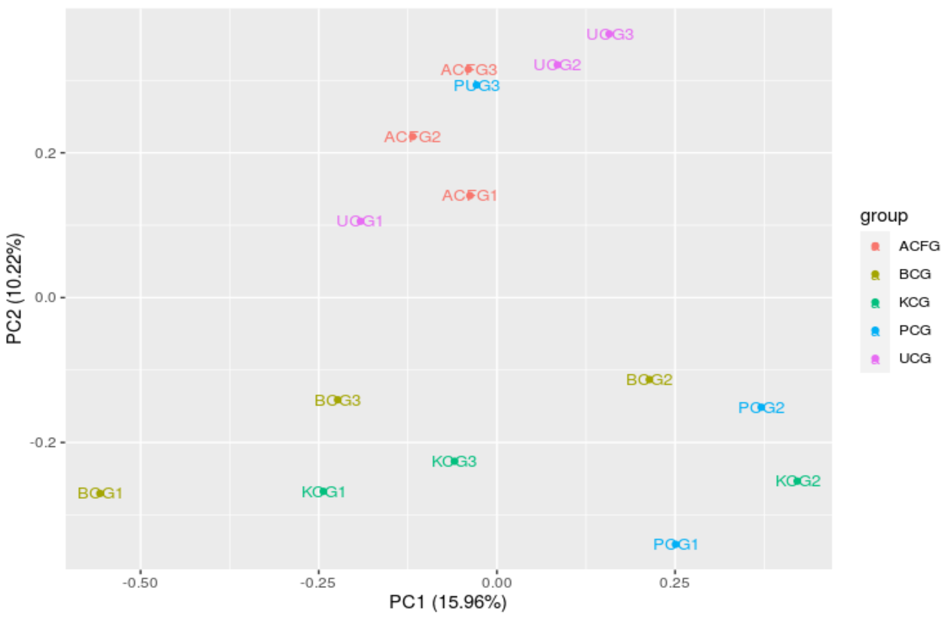

Supplementary Fig. 3. Principal component analysis of indigenous cattle breeds of Tamil Nadu based on deletions and duplications of CNV regions
