# Supplementary material for: Genome-wide copy number variation regions in indigenous (Bos indicus) cattle breeds of Tamil Nadu, India
Source: Anim Biosci. 2024 Aug 26;38(3):395–407. doi: 10.5713/ab.23.0525 (PMC11917407; doi:10.5713/ab.23.0525)
Supplement: Supplementary file 5 [file ab-23-0525-Supplementary-Table-S1.pdf]

Supplementary table 1. Chromosome-wise summary of CNVRs across cattle breeds of Tamil Nadu

Chr. no.	Chromosome Length (bp)	CNVR length (bp)	Per cent Length in CNVR	Number of CNVRs	Mean length (bp)	Median length (bp)	Minimum (bp)	Maximum (bp)
1	10,00,00,020	2,09,27,000	20.93	737	28,394.84	11,000	2,000	2,06,000
2	10,00,00,020	1,54,45,000	15.44	600	25,741.67	12,000	2,000	4,64,000
3	10,00,00,020	1,64,67,000	16.47	529	31,128.54	13,000	2,000	3,51,000
4	10,00,00,020	1,55,54,000	15.55	563	27,627.00	10,000	2,000	3,27,000
5	10,00,00,020	1,75,46,000	17.55	575	30,514.78	14,000	2,000	3,14,000
6	10,00,00,020	1,68,14,000	16.81	611	27,518.82	10,000	2,000	2,86,000
7	10,00,00,020	1,46,37,000	14.64	542	27,005.54	10,000	2,000	5,72,000
8	10,00,00,020	1,55,30,000	15.53	560	27,732.14	11,000	2,000	2,93,000
9	10,00,00,020	1,42,14,000	14.21	506	28,090.91	12,000	2,000	3,42,000
10	10,00,00,020	1,34,32,000	13.43	445	30,184.27	16,000	2,000	2,98,000
11	10,00,00,020	1,27,60,000	12.76	440	29,000.00	13,000	2,000	2,92,000
12	8,54,42,668	1,08,12,000	12.65	387	27,937.98	10,000	2,000	3,73,000
13	8,44,33,108	89,87,000	10.64	317	28,350.16	11,000	2,000	2,30,000
14	8,14,09,030	86,78,000	10.66	313	27,725.24	8,000	2,000	2,70,000
15	8,48,00,079	1,39,74,000	16.48	469	29,795.31	12,000	2,000	3,13,000
16	7,79,06,047	1,02,74,000	13.19	350	29,354.29	14,000	2,000	4,10,000
17	7,65,19,027	96,65,000	12.63	341	28,343.11	13,000	2,000	1,94,000

(Contd...)

Supplementary table 1. Chromosome-wise summary of CNVRs across cattle breeds of Tamil Nadu (Contd...)

Chr. no.	Chromosome Length (bp)	CNVR length (bp)	Per cent Length in CNVRs	Number of CNVRs	Mean length (bp)	Median length (bp)	Minimum (bp)	Maximum (bp)
18	6,59,48,792	95,69,000	14.51	342	27,979.53	11,000	2,000	2,44,000
19	6,53,17,831	80,42,000	12.31	276	29,137.68	15,000	2,000	1,84,000
20	7,58,62,604	93,48,000	12.32	338	27,656.80	11,000	2,000	2,55,000
21	6,93,07,409	91,72,000	13.23	330	27,793.94	10,000	2,000	4,22,000
22	6,18,92,534	59,75,000	9.65	236	25,317.80	11,500	2,000	1,72,000
23	5,33,31,160	68,18,000	12.78	237	28,767.93	13,000	2,000	2,26,000
24	6,50,17,658	73,32,000	11.28	268	27,358.21	12,000	2,000	2,10,000
25	4,40,44,299	57,75,000	13.11	197	29,314.72	9,000	2,000	3,05,000
26	5,18,61,174	72,68,000	14.01	241	30,157.68	11,000	2,000	1,92,000
27	4,87,49,331	71,58,000	14.68	241	29,701.24	13,000	2,000	2,45,000
28	4,61,05,673	51,65,000	11.20	206	25,072.82	11,000	2,000	2,17,000
29	5,21,31,593	82,20,000	15.77	262	31,374.05	10,000	2,000	2,18,000
X	88,516,652	858,76,000	97.02	134	6,40,866	23,9500	2,000	39,28,000
Y	39,421,065	391,77,000	99.38	12	32,64,750	20,500	2,000	3,03,70,000
Total	2,41,80,17,954	45,06,11,000	18.63	11,605	47,29,693	-	-	-
