# Supplementary material for: Genome-wide copy number variation regions in indigenous (Bos indicus) cattle breeds of Tamil Nadu, India
Source: Anim Biosci. 2024 Aug 26;38(3):395–407. doi: 10.5713/ab.23.0525 (PMC11917407; doi:10.5713/ab.23.0525)
Supplement: Supplementary file 6 [file ab-23-0525-Supplementary-Table-S2.pdf]

Supplementary table 2 Annotation of CNVRs in candidate genes for various phenotypic traits

Trait	Chromosome No.	Candidate genes
**Milk production related**		
305-day milk yield	1	*CACNA2D1, FBXO11, GRID2, LPIN1, MAP4K4, PKHD1* and *SLC24A2*
Lactation persistency	5	*TSPAN9*
	9	*MAP3K5*
	17	*IL-2*
	20	*C20H5ORF34*
Milk fat percentage	10	*ACO2, NFIB* and *NIPBL*
	11	*BRINP3, CDKN1A, EFR3A, EMP1, EPS8, ERCC6L2, GRIA4, ITSN2, MGST1, MYO16, NCKAP5, WDPCP, PHF20L1, SLC15A5, ST8SIA1, SUPT3H* and *SWT1*
	12	*BTN1A1, CD2, CPSF1, CRACR2A, CSN2, DERA, DPP10, MANSC1, PIK3C2G, PLCE1, RAI14, RNF19A, TLR4, TNFSF10, TTC33* and *VPS13B*
	13	*ARID5B, CSTF3, EPHA6, ERBB2, ITPR2, MATN2, MTMR12, RAB4A, SMCO3, SOX5, TBC1D1, KSR2, CDC42BPA, GRIN2B, PLCXD3, ZC3H3* and *ZNF484*
Milk fat yield	7	*SCD5*
	15	*CTNNA2, FAM168A, PPM1H, PTPN11, SLC4A4, SLC6A9* and *SUFU*
	16	*ANKRD44, CHST11, MAP6, TFCP2* and *USP24*
	17	*DCDC1, ERC1,* and *SHROOM3*
	18	*BEST3, C2CD3, LAMA4, PPME1, PRMT8* and *SUCLG2*
	19	*PARM1* and *POLD3*
	20	*ABCA1* and *ROBO1*
	21	*AFF2* and *SH3PXD2A*
	22	*ANO3, DIS3L2, IL2RB, LDLRAD3, METTL1* and *NELL2*
	23	*ARHGEF28, CNNM2* and *RASSF8*
Milk protein percentage	14	*IL-7*

(Contd...)

Supplementary table 2 Annotation of CNVRs in candidate genes for various phenotypic traits (Contd...)

Trait	Chromosome no.	Candidate genes
Milk yield	1	*DZIP3*
	3	*LAP3*, *AGBL4* and *BCAR3*
	4	*RBMS3* and *MFGE8*
	5	*ANO6, GALNT8*, *RPAP3*, *SPATS2* and *TULP3*
	6	*ADGRL3* and *RASSF6*
	9	*MOXD1*
	13	*MTG2*
	15	*FCHSD2*, *CDH13* and *MAML2*
	19	*KIF19* and *TOM1L1*
	22	*ERC2*
	23	*CDKAL1* and *ZFAND3*
	26	*TDRD1*
**Disease related**		
Somatic cell score	5	*SEC23A*
Clinical mastitis	2	*KIAA2012*
	8	*TLR4*
	9	*RPS6KA2*
	11	*KLHL29*
	14	*IL-7*
M. paratuberculosis susceptibility	1	*LPP* and *ZBTB20*
	2	*SLC11A1*
	5	*PPHLN1*, *STK38L* and *TMTC2*
	11	*TTC7A*
	23	*F13A1*
Tick resistance	9	*MYO6*
	16	*NR5A2*
	23	*DNAH8*

(Contd…)

Supplementary table 2 Annotation of CNVRs in candidate genes for various phenotypic traits (Contd...)

Trait	Chromosome no.	Candidate genes
Bovine respiratory disease susceptibility	7	*DPP6, EDNRB, IFT57, KCNH1, KCNQ3, MAPKAP1, PDE11A, SCD5, APBA2, CACNA1D, CHRNA7, DPYD, ERO1A, GLRA1, GPR158, LTA4H, PDE1A, RAD23B, STIM1, TBXAS1, TIMP2, TNRC6A, TRIP12, USP33, XBP1, YWHAB, ELOVL5, GUCY1A2, NR2F2, PDE9A, PLA2G4A, RGS7, SERPINC1, SH3RF1, SLC2A1, SMF1, TNRC6B, CACNB2, DRD2, ELOVL7, FADS2, IMPACT, INSIG1, KCNJ3, KCNMA1, PRKCI, PRKG1 and SLC6A3*
Bovine tuberculosis susceptibility	5	*SEC31A*
Digital dermatitis	7	*OLFM3*
FMD susceptibility	7	*DDX10*
**Adaptability related**		
Heat tolerance	1	*SOD1*
Body temperature	1	*ASL* and *CAST*
Oxidative stress response	3	*PLCB1* and *AOX1*
Residual feed intake	14	*SMARCAL1*
	15	*AOX1, ASNSD1, CCSER1, CDH13, HMCN1, PQLC2, MYO10, RARRES2, REEP3* and *RUFY3*
Thermal stress	17	*IL-2*
**Growth related**		
Meat production	5	*CCDC141*
Body weight	6	*LDB2*
	7	*NPC1*
	29	*HGF*
	22	*EGFR*
Insulin-like growth factor 1 level	28	*NKAIN3*

(Contd...)

Supplementary table 2 Annotation of CNVRs in candidate genes for various phenotypic traits (Contd...)

Trait	Chromosome no.	Candidate genes
**Meat and carcass related**		
Meat tenderness	9	*MAP3K5*
Fat percentage	13	*FTO*
Inter muscular fat percentage	13	*BCL6*
Kidney, heart fat percentage	13	*NCAM1*
Meat tenderness	17	*ITGA1*
Subcutaneous fat thickness	28	*EPS15L1*
**Reproduction related**		
Fertility index	15	*BRWD1* and *PDE6D*
Calving ease	2	*GADL1, RBM47, ROR1, SART3, SLC24A4, SLC8A3, SYT3, TCF20, ADAMTS17, CCDC91, CEP70, COL4A6, COL12A1, CSGALNACT1* and *PRKD1*
	3	*ACSS3, CUL3, ZNF407, EIF2B3, ENTHD1, FAM134B, LAP3, NALCN, NBAS, PLCB1, USP13, ARHGAP10, CTNNA3, DOCK1, EBF1, FHIT, HHAT, HPCAL1, IGSF5, KCNC3, KDM4C, KLHL1, NRG3, PHF2, TBC1D12* and *TRABD2B*
	4	*ABCC9, ARHGAP22, GRIA3, MND1, RASGRP3, SHANK2, CRTC1, FRMD5, GRIK2, LRBA, MAML3, MAN2A1, MFGE8, RBMS3, SLC10A7, SLIT3* and *WDFY3*
	5	*ABCC1, ARSG, CCDC141, CNTN5, CTU1, DOK6, DOCK5, DSCAM, KIF26B, MBNL3, SERPINB5, ADCY5, AKAP6, ARHGAP26, CSAD, ESR1, HSD17B12, OSR1, PLCH1, RCAN2, RPS6KC1* and *SLCO1A2*
**Exterior traits**		
Teat Placement	4	*WDFY3*
Hair length	5	*OSR1* and *PCCA*
Feet and leg conformation	6	*CRH, NTM, RASGRF1, CDH6, DHX35, FLNB, OSBPL10, PAK5, PITRM1, PRDM16, SNX29, SRGAP2* and *XRCC4*
	7	*PRKAG3, C10H15ORF41, DHX36, DTNB, GRB2, MAGI1, MID1, OTOP3* and *RASA3*
Rump conformation	5	*OTOA* and *PPM1B*
	7	*ANK1*
Eye area pigmentation	13	*ATRN*
	22	*MITF*
