# Supplementary material for: Genome-wide copy number variation regions in indigenous (Bos indicus) cattle breeds of Tamil Nadu, India
Source: Anim Biosci. 2024 Aug 26;38(3):395–407. doi: 10.5713/ab.23.0525 (PMC11917407; doi:10.5713/ab.23.0525)
Supplement: Supplementary file 7 [file ab-23-0525-Supplementary-Table-S3.pdf]

Supplementary Table 3. Details of criteria followed for pooling of DNA samples in five cattle breeds of Tamil Nadu

Breed	Group	Category	Milk yield (kg)	No. of animals pooled	Concentration (ng/µl)	A260/A280
**Alambadi**	ACG 1	Bulls	-	2	209.5	1.89
	ACG 2	Farm samples	-	7	203.4	1.88
	ACG 3	Field samples	-	5	207.1	1.85
**Bargur**	BCG1	Bulls	-	5	207.3	1.80
	BCG2	Medium yielders	>600 kg/lactation	7	208.1	1.83
	BCG3	Low yielders	<300 kg/lactation	7	212.8	1.84
**Kangayam**	KCG1	Bulls	-	10	206.5	1.82
	KCG2	Medium yielders	>2 kg/day	4	206.7	1.84
	KCG3	Low yielders	<1 kg/day	2	207.2	1.87
**Pulikulam**	PCG1	Bulls	-	2	210.1	1.84
	PCG2	Medium yielders	>1 kg/day	7	209.2	1.86
	PCG3	Low yielders	<1 kg/day	7	209.0	1.86
**Umblachery**	UCG 1	Bulls	-	6	202.8	1.87
	UCG 2	Medium yielders	>2 kg/day	4	202.4	1.87
	UCG 3	Low yielders	<1 kg/day	4	191.1	1.87
**Total**				**79**		
