# Supplementary material for: Genome-wide copy number variation regions in indigenous (Bos indicus) cattle breeds of Tamil Nadu, India
Source: Anim Biosci. 2024 Aug 26;38(3):395–407. doi: 10.5713/ab.23.0525 (PMC11917407; doi:10.5713/ab.23.0525)
Supplement: Supplementary file 8 [file ab-23-0525-Supplementary-Table-S4.pdf]

Supplementary Table 4. Details of primers used for validation of copy number variation regions

Gene	Breed identified for CNVR	Primers (5' to 3')	Chromosome No.	CNVR type	Location in the genome		Size of amplicon (bp)
					From (bp)	To (bp)	
**BTF3**	-	FP: CCG TAA TGG TGA AAG TGT TTG C<br>RP: ACA GGT TGC TTA GAC TTC TGC T	20	Normal	84,82,935	84,83,124	190
**PRKG1**	Alambadi	FP: ATG TGT GTC ACT GAT ACG AAT G<br>RP: GGA AGA TGT GAG AAC AGC G	26	Deletion	73,39,101	73,39,219	119
**GLDC**	Bargur	FP: GTG CTG TTC CAG TAC CCA GAC<br>RP: AGA CAC TCC TCC ACG GAC C	8	Duplication	403,63,801	403,63,920	120
**RERE**	Kangayam	FP: AAG AAC CAG TTG GGG GAT G<br>RP: ATC ACT CAG CAG ACC AGT G	16	Deletion	420,41,644	420,41,799	156
**KIF11**	Pulikulam	FP: GAC AAC CGC AGC AAC TCT GA<br>RP: CTT GAC ACG CCC CAA TGT GA	26	Duplication	145,98,525	145,98,647	123
**FBXO40**	Umblachery	FP: GAT CTG ACT GCT CAA AAA TGC C<br>RP: CAA CCA GTG CTA GTG AGA GTC	1	Deletion	671,90,078	671,90,311	234
