# Supplementary material for: Genome-wide copy number variation regions in indigenous (Bos indicus) cattle breeds of Tamil Nadu, India
Source: Anim Biosci. 2024 Aug 26;38(3):395–407. doi: 10.5713/ab.23.0525 (PMC11917407; doi:10.5713/ab.23.0525)
Supplement: Supplementary file 4 [file ab-23-0525-Supplementary-Fig-S5.pdf]

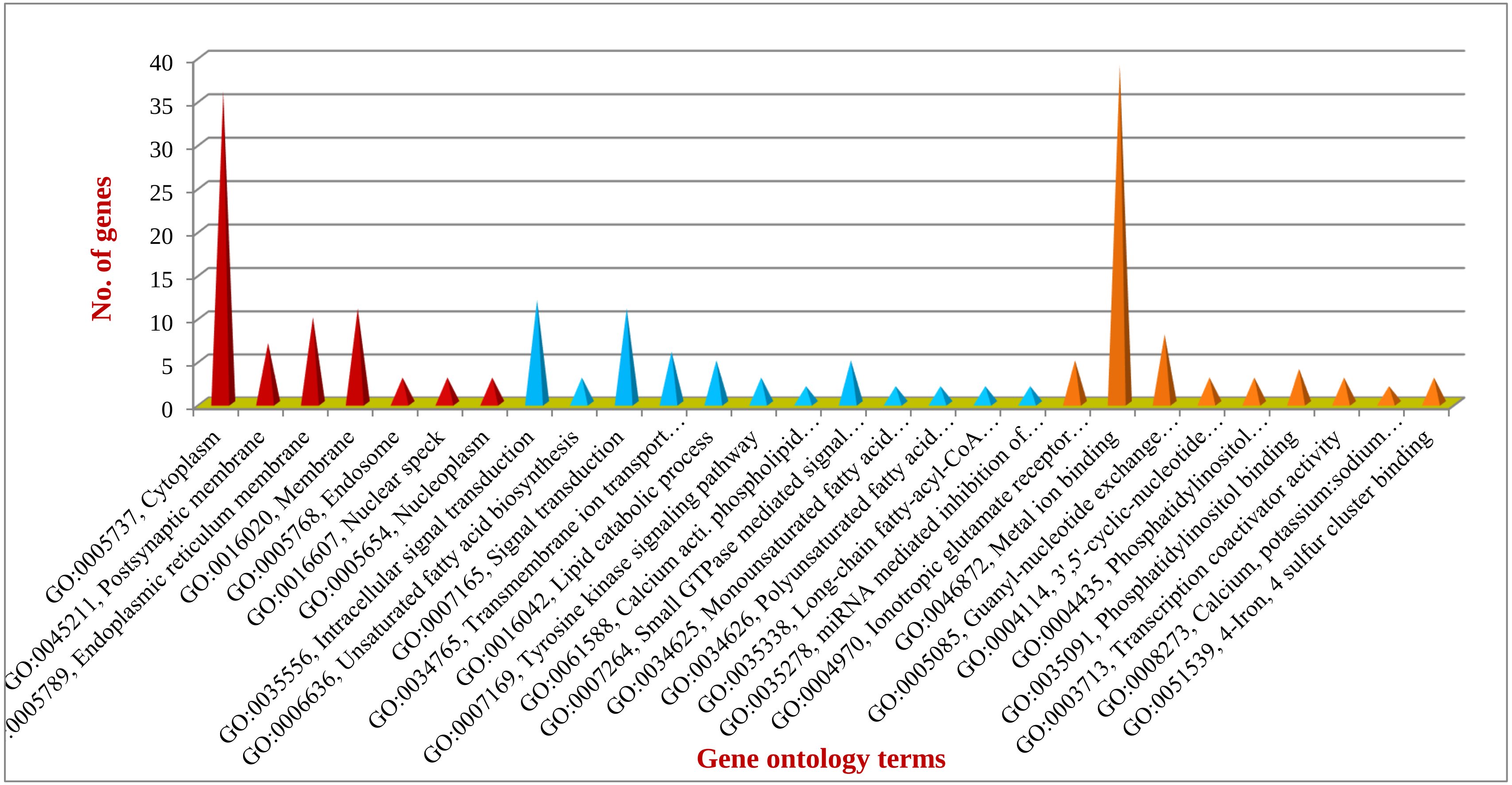


**Supplementary Fig. 5 Gene ontology terms of candidate genes**

(Red cones: cellular components; Blue cones: Biological processes; Orange cones: Molecular functions)
